# Identification of Barriers to Digital Patient Portal Adoption Among Patients With Prostate Cancer Undergoing Curative Radiotherapy at a Canadian Provincial Cancer Program: A Quality Improvement Study

**DOI:** 10.1200/CCI-25-00162

**Published:** 2026-04-06

**Authors:** Nikunj Patil, Rashmi Koul, Jennifer Moyer, Ian Kudel, Jennifer Beaudry, Annette Christianson, Julian O. Kim

**Affiliations:** ^1^ Section of Radiation Oncology, Department of Radiology, Max Rady College of Medicine, University of Manitoba, Winnipeg, MB, Canada; ^2^ Department of Radiation Oncology, CancerCare Manitoba, Winnipeg, MB, Canada; ^3^ Department of Radiation Therapy, CancerCare Manitoba, Winnipeg, MB, Canada; ^4^ Varian, A Siemens Healthineers Company, Palo Alto, CA; ^5^ Paul Albrechtsen Research Institute, CancerCare Manitoba, Winnipeg, MB, Canada

## Abstract

**PURPOSE:**

Digital patient portals (DPPs) provide patients a direct electronic link with their care teams allowing secure, rapid symptom reporting and the capture of electronic patient-reported outcome questionnaires. A proportion of patients face barriers to DPP adoption. We conducted a prospective observational cohort study to quantify the proportion of DPP nonadopters and identify factors associated with DPP nonadoption.

**METHODS:**

All patients with prostate cancer undergoing radical radiotherapy at a Canadian Provincial Cancer Program were given the opportunity to install a DPP on their mobile device/tablet before starting radiotherapy. Each patient completed a self-reported questionnaire assessing their willingness and ability to use the DPP and quantified variables associated with their decision. A DPP adopter was defined as a patient who logged into the application and accessed their radiotherapy educational materials. Descriptive variables were tabulated by DPP adoption status. Differences in distributions of baseline characteristics were assessed using standard parametric and nonparametric statistics. Univariable and multivariable logistic regression analyses were performed to identify variables associated with DPP nonadoption.

**RESULTS:**

Between July 2022 and January 2023, 118 patients with prostate cancer were enrolled with a median age of 71 years (range, 50-86). Most patients (56.8%) had a college diploma, 29.6% lived rurally, and 95.8% owned a smartphone. Twenty-eight patients (23.8%) were DPP nonadopters. On stepwise multivariable logistic regression analysis, poor self-reported ability to use mobile devices was associated with DPP nonadoption (*P* < .01).

**CONCLUSION:**

Among patients with prostate cancer undergoing radical radiotherapy, poor self-reported ability to use smartphone technology was identified as the primary barrier to DPP adoption. Interventions geared at mitigating this barrier to DPP adoption are needed.

## INTRODUCTION

Digital patient portals (DPPs) are valuable tools in various health care settings. DPPs typically consist of an electronic application installed on patient's personal device (smartphone, tablet), which is linked to their electronic medical record system (EMR).^1^ DPPs empower patients to actively engage in their health care journey and foster better communication with their health care team allowing patients to report symptoms and obtain prompt management of symptomatic concerns.^2,3^ This has been shown to result in fewer emergency department visits for oncology patients.^4,5^ There is also growing evidence that patients are more willing to disclose and seek help for psychological issues using DPPs.^6^

**CONTEXT**

**Key Objective**
To identify barriers to adoption of digital patient portals (DPPs) among patients with prostate cancer receiving radical radiotherapy in a Canadian provincial cancer program.
**Knowledge Generated**
This study identified that in a contemporary setting, the DPP nonadoption rate is 23.8%. Although most patients had access to internet and smartphones, the primary barrier to the adoption of a DPP was identified to be poor self-reported ability to use mobile smartphone technology.
**Relevance *(Z. Bakouny)***
DPP adoption can improve patient access to care by allowing them to more easily schedule appointments, communicate with their care teams, and obtain prompt care. This study investigated barriers to adoption of DPPs and found that the most predictive factor for non-adoption was poor self-reported ability to use a smartphone.*
*Relevance statement written by *JCO CCI* Associate Editor Ziad Bakouny, MD, MSc.

DPPs can collect quality-of-life (QoL) information from patients including electronic patient-reported outcomes (ePROs). DPPs allow accessible and real-time collection of these parameters.^7,8^ These digital interventions are associated with better emotional and physical functioning, improved QoL, decreased symptom burden, and less distress.^9-12^ Moreover, DPPs provide patients access to their EMRs, which improves patient self-management^13,14^ and their relationship with health care professionals (HCPs).^15,16^ Because of the advantages of DPPs, establishing and providing patients access to their EMRs is a pivotal part of the World Health Organization's global strategy for digital health.^17^ Finally, self-scheduling tools within DPPs empower patients to reschedule appointments and imaging studies, thereby reducing administrative burden on health care administrative staff, facilitating more efficient care.^18,19^

Despite the advantages of DPPs, barriers to their adoption remain. Factors such as ethnicity, language, advanced age, male sex, low socioeconomic status, and poor literacy have been associated with suboptimal adoption of DPPs.^20-22^ Other factors like development of clinical workflow and training of health care workers in DPP use have been associated with improved uptake of DPPs.^23^ DPPs became important channels for timely health care access for patients during the SARS-COV-2 lockdowns. As a result of the SARS-COV-2 pandemic, digital tools are not just for early-adopting patients but are now central to how health care services are delivered to whole populations.^24-26^ There is therefore a *bone fide* need to address the root causes of disparities of access to DPPs within different populations and implement strategies to mitigate barriers to their use.

CancerCare Manitoba (CCMB) is the sole source, publicly funded, provider of oncology services to the universally insured population of the Canadian Province of Manitoba, with a catchment of 1.4 million persons. Noona (Varian, a Siemens Healthineers Company) is an oncology-focused DPP which connects patients (via a patient's smartphone/tablet) with their oncology team and the ARIA EMR at CCMB. Noona is a validated tool for ePRO collection.^27^ In 2021, Noona was introduced for clinical use at CCMB as a symptom-reporting tool for patients, a repository for patients to access educational materials pertaining to their oncologic care and to collect ePROs directly from patients/family members. Among the various patient populations given access to Noona, patients with prostate cancer were considered a priori at higher risk of DPP nonadoption because of a higher prevalence of known barriers to DPPs in this population including advanced age, male sex, and some subgroups with lower SES or literacy levels. Anticipating a modest degree of DPP nonadoption among patients with prostate cancer, we conducted a prospective quality improvement study designed to assess the proportion of patients with prostate cancer who were unwilling to use DPPs and to assess which patient, treatment, or other factors were associated with nonadoption, with the goal of informing a future knowledge translation intervention to reduce DPP nonadoption.

## METHODS

### Study Design

This prospective quality improvement study followed the plan-do-study-act framework for study design. In the preintervention phase of the study, Phase 1 (P1), the aim was to quantify the degree of DPP nonadoption among patients with prostate cancer receiving radical radiotherapy at CCMB and determine which factors were associated with DPP nonadoption. The data gathered during P1 will be used to develop a custom intervention aimed at improving DPP adoption in the future. After implementation of the intervention, phase 2 of the study (P2) consists of measuring the impact of the intervention on the DPP nonadoption rate and assessment of factors associated with persistent nonadoption.

For the present study, P1, all patients with prostate cancer undergoing radical radiotherapy at CCMB were offered voluntary participation in the study at their computed tomography (CT) simulation scan appointment or radiation patient education appointment by study investigators. Consented participants completed a comprehensive self-reported questionnaire consisting of 18 questions (Data Supplement, Appendix A) which assessed a range of variables including demographics, proficiency with computing and mobile technology, previous experience with DPPs, and willingness (yes *v* no) to use the DPP application during their care and follow-up. DPP nonadopters were also queried regarding their willingness to participate in in-person or online tutorial sessions to facilitate DPP adoption. Patients unwilling to use the DPP were allowed to use a pen and paper to complete the study questionnaire. All patients received a radiation therapy educational package containing information about their upcoming radiation therapy along with instructions on bowel/bladder preparation (Data Supplement, Appendix B). The educational package was sent out electronically using the DPP for DPP adopters but was given out on paper for DPP nonadopters. There was no patient out-of-pocket expense associated with the use of the DPP, and the app was paid for by CCMB.

### Statistical Considerations

Patient characteristics from the questionnaire were tabulated by DPP adoption category (yes *v* no), and standard parametric and nonparametric statistical tests including the student t-test, chi-squared test, or z-test were used to assess for significant differences in distributions of baseline characteristics. A DPP adopter was defined as a patient who both logged into the application and viewed their radiotherapy educational materials. DPP nonadopters were defined as those who neither logged into nor viewed their radiotherapy treatment teaching materials in the DPP. Univariable logistic regression analysis was performed to test for associations between DPP adoption (yes *v* no) and potential explanatory variables obtained from the patient questionnaire. Two multivariable logistic regression models were built. The first multivariable logistic regression model included all variables with univariate logistic regression *P* values <.2. The second multivariable stepwise logistic regression model was built using the Akaike information criterion (AIC) method to evaluate the goodness of model fit and maximize model parsimony. For our analysis, ordinal data were considered linear in the logistic regression modeling. For purposes of this analysis, the a priori level of statistical significance (α) was set to .05.

### Sample Size

We assumed a DPP nonadoption rate of 15% during P1. Assuming that the future planned intervention would reduce the nonadoption rate by 50% (relative risk reduction), a sample size of N = 216 (N = 108 in P1 and N = 108 in P2) would yield a power of 80% with an alpha error of 0.05 with a one-sided test for significance. Therefore, a minimum of N = 108 were planned to be assessed in P1 before implementing the intervention for P2.

### Ethics Approval

This prospective study involving human participants was performed in accordance with the ethical standards of the institutional research committee and with the Declaration of Helsinki 1964 and its later amendments or comparable ethical standards. The University of Manitoba Research Ethics Committee (approval No.: HS26354) and CCMB Research Resource Impact Committee (approval No.: 2024-02) approved this study. All study participants provided voluntary written consent before partaking in any study-related activities.

## RESULTS

### Patient Demographics

Between July 2022 and January 2023, all patients with prostate cancer planned for radical radiotherapy were approached for participation (N = 141), among which 118 patients consented to participate in the study. The demographic characteristics of the cohort are summarized in Table 1. The mean age was 70.1 years (range, 50-86 years). The mean age of nonadopters was higher compared with that of adopters (72.3 *v* 69.5 years); however, this was not statistically significant. The majority of the cohort were married (78.8%) and were urban dwellers (70.3%) with no significant differences between adoption groups. White patients (83.4%) were the most common ethnic group, followed by First Nations (5%) and African American (5%) patients. More than half of patients (56.6%) had a college diploma. There was a significant difference (*P* < .001) in the annual household income between adoption groups whereby 58.9% of the DPP adopters had incomes above $43,801 Canadian dollars (CAD) versus 21.5% of the nonadopters. There was no significant difference in self-reported health status between adoption groups.

**TABLE 1.** Baseline Demographic Characteristics of the Cohort

Characteristic	Whole Cohort (N = 118)	DPP Adopters (n = 90)	DPP Nonadopters (n = 28)
Age (mean, range)	70.1 years (50-86)	69.5 years (50-86)	72.3 years (59-82)
Marital status, No. (%)			
Married or common law	93 (78.8%)	71 (78.9%)	22 (78.6%)
Single or divorced	25 (21.2%)	19 (21.1%)	6 (21.4%)
Ethnicity, No. (%)			
White	98 (83.4%)	71 (78.9%)	27 (96.4%)
First nations or aboriginal	6 (5%)	5 (5.6%)	1 (3.6%)
Asian	6 (5%)	6 (6.7%)	0
African American	3 (2.5%)	3 (3.3%)	0
Hispanic	2 (1.6%)	2 (2.2%)	0
Unknown	3 (2.5%)	3 (3.3%)	0
Place of dwelling, No. (%)			
Urban	83 (70.3%)	64 (71.1%)	19 (67.9%)
Rural	35 (29.7%)	26 (28.9%)	9 (32.1%)
Education level, No. (%)			
Less than high school	13 (11.2%)	6 (6.7%)	7 (25.0%)
High school or equivalent	38 (32.2%)	27 (30.0%)	11 (39.3%)
College diploma	34 (28.8%)	28 (31.1%)	6 (21.4%)
University degree	25 (21.1%)	21 (23.3%)	4 (14.3%)
Masters or doctorate	8 (6.7%)	8 (8.9%)	0
Annual household income,^a^ No. (%)			
<$30,600 CAD	11 (11.8%)	6 (6.7%)	5 (17.9%)
$30,601 CAD to $43,800 CAD	16 (13.5%)	6 (6.7%)	10 (35.7%)
$43,801 CAD to $57,100 CAD	16 (13.5%)	15 (16.7%)	1 (3.6%)
$57,101 CAD to $76,100 CAD	15 (12.6%)	10 (11.1%)	5 (17.9%)
>$76,100 CAD	28 (23.7%)	28 (31.1%)	0
Prefer not to say	32 (27.1%)	25 (27.8%)	7 (25%)
Language, No. (%)			
English	114 (96.6%)	87 (96.7%)	27 (96.4%)
Other	4 (3.4%)	3 (3.3%)	1 (3.6%)
Self-reported health status, No. (%)			
Excellent	5 (4.2%)	5 (5.6%)	0
Very good	14 (11.8%)	10(11.1%)	4 (14.3%)
Good	59 (50%)	45 (50%)	14 (50%)
Fair	33 (27.9%)	25 (27.8%)	8 (28.6%)
Poor	6 (5%)	4 (4.4%)	2 (7.1%)
Unknown	1 (1.1%)	1 (1.1%)	0

Abbreviations: CAD, Canadian dollars; DPP, digital patient portal.

a Canadian dollars.

### Access to Technology

The majority of the cohort (95.7%) had access to a tablet/smartphone, and 94% had internet at home, with no difference in access to devices between adopter groups. However, the proportion of patients with access to mobile data (Long Term Evolution or 4G) was greater among DPP adopters compared with nonadopters (81.1% *v* 53.6%, *P* < .01). Daily internet usage was reported in 83% of the cohort, for which there was no significant difference by adoption group (*P* = .10). The internet connectivity and technological habits of the cohort are summarized in Table 2.

**TABLE 2.** Internet Connectivity and Technologic Habits

Characteristic	Whole Cohort (N = 118), No. (%)	DPP Adopters (n = 90), No. (%)	DPP Nonadopters (n = 28), No. (%)
Access to smartphone or tablet			
Yes	113 (95.7%)	86 (95.6%)	27 (96.4%)
No	5 (4.3%)	4 (4.4%)	1 (3.6%)
Access to home internet			
Yes	111 (94%)	85 (94.4%)	26 (92.9%)
No	7 (6%)	5 (5.6%)	2 (7.1%)
Access to mobile data (LTE/4G)^a^			
Yes	88 (74.5%)	73 (81.1%)	15 (53.6%)
No	29 (24.5%)	16 (17.8%)	13 (46.4%)
Missing	1 (1%)	1 (1.1%)	0
Frequency of internet use			
Daily	98 (83%)	78 (86.7%)	20 (71.4%)
Weekly	10 (8%)	7 (7.8%)	3 (10.7%)
Monthly	1 (1%)	0	1 (3.6%)
Never/missing	9 (8%)	5 (5.6%)	4 (14.3%)
Self-rated smartphone ability			
Excellent	7 (5.9%)	6 (6.7%)	1 (3.6%)
Very good	19 (16.1%)	17 (18.9)	2 (7.1%)
Good	32 (27.2%)	29 (32.2)	3 (10.7%)
Fair	40 (33.9%)	28 (31.1%)	23 (42.9%)
Poor	20 (16.9%)	10 (11.1%)	10 (35.7%)
Confidence with medical questionnaires			
Extremely confident	14 (12%)	14 (15.6%)	0
Very confident	24 (20.3%)	21 (23.3%)	3 (10.7%)
Confident	59 (50%)	41 (45.6%)	18 (64.3%)
Somewhat confident	16 (13.5%)	11 (12.2%)	5 (17.9%)
Not confident	5 (4.2%)	3 (3.3%)	2 (7.1%)
Confidence with privacy safeguards			
Completely confident	61 (51.6%)	44 (48.9%)	17 (60.7%)
Fairly confident	37 (31.6%)	34 (37.8%)	3 (10.7%)
Slightly confident	6 (5%)	11 (12.2%)	3 (10.7%)
Somewhat confident	14 (11.8%)	1 (1.1%)	5 (17.9%)
Prior use of DPP			
Yes	22 (18.6%)	21 (23.3%)	1 (3.6%)
No	76 (81.4%)	69 (76.7%)	27 (96.4%)

Abbreviations: DPP, digital patient portal; LTE, long-term evolution.

a 4G = fourth-generation wireless data.

### Proficiency Using Digital/Mobile Technology

There was variability within the cohort pertaining to their self-reported ability to use a smartphone or mobile device. Among DPP nonadopters, 35.7% rated their ability to use a smartphone as poor versus 11.1% among DPP adopters (*P* < .01). The majority of the cohort (82.3%) reported that they felt confident answering medical questionnaires independently without any statistically significant difference in confidence by the adoption group. The use of DPPs in this patient population was a novel experience for the majority of the cohort, whereby only 18.6% had a history of using DPPs for other medical encounters, consisting of 3.6% of the nonadopters and 23.3% of the adopters (*P* = .01). These findings are summarized in Table 2.

### Willingness to Use a DPP

Of the 118 patients included in the study who were offered the opportunity to use the DPP, we found that 23.8% (n = 28) chose not to adopt the DPP as part of their oncology care. Among nonadopters, 21.4% was interested in learning more about using a DPP through either an in-person tutorial or an online video tutorial. Nonadopters were asked to self-report their primary reason for DPP nonadoption (Data Supplement, Table S1). Reasons for nonadoption include preference for pen and paper (46.4%), self-reported personal disorganization (14.2%), feeling overwhelmed (7.2%), lack of confidence in security of personal health information on digital platforms (7.2%), or mixed reasons (14.2%).

### Factors Associated With DPP Nonadoption: Univariate Logistic Regression Model

In the univariable regression (Table 2), patients with better self-reported ability to use a smartphone/tablet were less likely to be nonadopters. For example, patients with good self-reported ability to use a smartphone had lower odds of DPP nonadoption (odds ratio [OR], 0.22 [95% CI 0.09 to 0.58]) compared with those with poor self-reported ability to use a smartphone. Patients with education levels less than high school had higher odds of DPP nonadoption (OR, 5.72 [95% CI 1.6 to 20.7]) compared with those who completed high school or higher.

Household income level was associated with DPP nonadoption whereby for each increase in income strata level, odds of DPP nonadoption decreased (OR [per unit increase in income strata level], 0.45 [95% CI, 0.29 to 0.69]). Confidence in answering medical questionnaires was also associated with DPP nonadoption whereby patients who felt confident had lower odds of nonadoption (OR, 0.27 [95% CI, 0.10 to 0.72]) compared with those who felt not at all confident answering medical questionnaires independently.

### Factors Associated With DPP Nonadoption: Multivariable Logistic Regression Model

The first multivariable model (Table 3) was built using a variable selection process whereby all covariates with univariable *P* < .2 were all entered into a standard multivariable logistic regression model. This model found that two variables were significantly associated with DPP nonadoption after adjusting for other variables in the model. First, White ethnicity had higher odds of DPP nonadoption compared with non-White patients (adjusted OR [aOR], 54.8 [95% CI, 2.51 to >999.99]), and increased levels of self-reported confidence answering medical questionnaires were associated with lower levels of DPP nonadoption (aOR [per unit increase in confidence scale], 0.46 [95% CI, 0.21 to 0.99]). However, given the wide confidence intervals of the OR of the White ethnicity category, this association is attributable to the small number of participants of non-White ethnicity in the nonadopter group.

**TABLE 3.** Standard Multivariable Logistic Regression for DPP Nonadoption

Variable	Odds Ratio	95% CI	*P*
Age (per year increase)	0.98	0.89 to 1.07	.61
Education level			
Less than high school	4.70	0.63 to 35.42	.46
High school or equivalent	1.83	0.58 to 5.81	
Masters/doctorate	<0.001	<0.001 to >999.9	
College/university	Ref	Ref	
Ethnicity (White vs others)	54.88	2.5 to >999.9	.01
Access to mobile data (yes *v* no)	0.14	0.02 to 1.17	.07
Composite score of access to device, internet, and mobile data^a^	2.89	0.56 to 15.02	.21
Frequency of internet use (per Likert scale unit)	0.73	0.32 to 1.69	.47
Ability to use a smartphone (per Likert scale unit)	0.78	0.41 to 1.50	.46
Confidence in answering medical questionnaires alone (per liker scale unit)	0.46	0.21 to 0.99	.05
Prior experience using digital portals (yes *v* no)	0.13	0.01 to 1.16	.07

Abbreviation: DPP, digital patient portal.

a Per unit increase in ordinal scale value.

The second stepwise logistic regression model using the AIC was built to remove colinear variables and improve the statistical stability of the model. The AIC multivariable model found that self-reported confidence using a smartphone or mobile device was the only variable which was significantly associated with DPP nonadoption. Patients with good self-reported ability to use a smartphone or mobile device had reduced odds of nonadoption (aOR, 0.22 [95% CI 0.09 to 0.58]) compared with those who reported poor ability to use a smartphone or mobile device. This corresponds to an adjusted OR of 0.47 (95% CI, 0.29 to 0.76) per unit increase on the ordinal scale of self-reported confidence using a smartphone/tablet (Table 4).

**TABLE 4.** Stepwise Multivariable AIC Logistic Regression for DPP Nonadoption

Variable	Odds Ratio	95% CI	*P*
Ability to use a smartphone^a^	0.47	0.30 to 0.76	<.01

Abbreviations: AIC, Akaike information criterion; DPP, digital patient portal.

a Per unit increase in ordinal scale value.

## DISCUSSION

The advent of digital applications which directly connect patients with care teams and their electronic medical record via a wireless mobile or internet-connected devices has come with significant opportunities and challenges. Adoption behavior within populations has been long recognized to follow along strata lines, as first described in 1903 by Gabriel Tarde in the field of implementation science as the diffusion of innovation theory.^28^ This model, and its derivatives, describes how, in response to innovations, adoption within a population will occur first among innovators and early adopters, later with the early majority who adopt if the innovation is practical and has mass appeal, and finally by those who are initially skeptical or change adverse but who adopt when peers or necessity compels them to do so. Given the significant advantages of DPPs for patients and their caregivers, DPP nonadoption represents a disparity in care, one which is imperative to minimize.

Our study found that among 118 patients with prostate cancer undergoing radical radiotherapy in a contemporary clinical oncology setting, 23.8% of patients chose not to adopt a DPP. We aimed to identify factors associated with their nonadoption to potentially offer means to ultimately facilitate adoption.

The univariate logistic regression found that nonadoption was associated with poor self-reported ability to use smartphones, lack of access to mobile data, low frequency of internet use, lack of confidence in answering medical questionnaires, low educational level, low income level, and lack of previous history of using DPP. The forward selection stepwise linear regression found that patient self-reported ability to use mobile computing technology such as a smartphone or a tablet was the most significant predictor and that no other variable was significant when added to the model. Thus, based on these responses, a patient's self-reported ability to use mobile computing technology is the primary driver of respondents' willingness to use DPP. The findings of this study are important in that it has identified a *potentially modifiable* barrier to adoption for which knowledge translation efforts can be focused on so to increase DPP adoption.

Among prostate cancer cohorts, Appleyard and colleagues^33^ evaluated the feasibility of using ePROs in men with advanced prostate cancer and found that 88% (40 patients out of 45) of patients were willing use them. This adoption rate was somewhat higher than our study perhaps owing to the fact that ePROs were completed on-site at first, with the option of remote completion at 3 months. At 3 months, their on-site adoption was 87% (35 patients of 40); however, the rate of remote completion was only 24%. They attributed their poor remote completion rate to various factors such as fatigue, concerns about downloading an application, and process-based problems (issues with usernames and/or password). A study by O'Connor et al^34^ assessed the acceptability and usability of DPPs in patients with prostate cancer across five NHS hospitals and found that 60% of patients with prostate cancer were willing to try the DPP, but only 37% of patients logged in over a 6-month period. The most common reasons for this nonadoption included lack of access to a computer, not wanting to use computers for health care purposes, a dislike of computers, and lack of internet access.

Griffin et al^21^ evaluated disparities in DPP access among 28,942 patients with cancer with a mix of various primary tumor types and found that males, racial and ethnic minorities, rural dwellers, and those with limited internet access had lower odds of using DPPs. Several other studies have emphasized that advanced age, racial minority status, and low educational levels are associated with DPP nonadoption.^2,35-38^ The French FASTOCH study^22^ estimated the feasibility of using DPPs in patients who were older than 75 years and found that 60.8% of patients in that age category were not willing to use DPPs. The most common reason for nonadoption was lack of access to the internet or uneasiness with technology. On univariable and multivariable analyses, they found that patients were willing to use DPPs if they had a higher socioeconomic status and if they had good self-reported health status.

Rodler and colleagues^39^ reported a declining prevalence of digital technology use with advancing age in patients with advanced genitourinary cancers in the era before the SARS-COV-2 pandemic. The use of smartphones and internet declined after age 70 years and among those age 80 to 89 years, with only 38% reported using mobile devices. A Statistics Canada General Society survey with data collected from November 2018 until March 2019 which were published in 2021 on the evolving use of internet among Canadians and found that only 9% of participants were internet nonusers.^40^ However, the proportion of internet nonusers was 28.8% among participants 65 years or older. The survey concluded that digital use habits are, in part, explained by advanced age, poor educational qualification, low socioeconomic level, and employment status. Given the rapid adoption of mobile internet technology in our population over the past decade, it is no surprise that among our study population (which was studied in 2022 and 2023), we found that 94% had access to the internet at home, and there was also heightened digital reliance after the COVID-19 pandemic, which also likely contributed to our patient population having relatively high internet access at home.^41,42^ However, home internet access and digital literacy do not necessarily guarantee the ability to confidently use a DPP. Design complexity, unfamiliarity with digital tools, lack of confidence, and troubleshooting difficulties are all barriers to DPP adoption. Many users also fear making mistakes or being unable to get assistance when needed.^43,44^ Addressing these concerns through personalized solutions is essential for improving adoption.

Our study has several notable strengths. Our sample population was representative and contemporary and captured key factors such as ethnicity, educational status, annual income, area of living, and their first language, which have been shown to have an impact on adoption of DPPs in the literature. The sociodemographic distribution of patients with prostate cancer included in our study parallel those of a typical Canadian provincial prostate cancer population. Saad et al^45^ evaluated the epidemiologic trends for patients with prostate cancer in Ontario and found that the mean age for nonmetastatic patients was 67.4 years, with 14% living in rural areas and 33.6% with an annual income within the second quantile. These findings correspond with our cohort for which the mean age was 70.1 years, with 29.7% living in rural areas and 38.8% had an annual income less than $57,100 CAD. Our study also addressed a gap in the existing literature for this milieu, evaluating the technological habits of participants and identifying the key barrier to adoption.

Our study limitations include our inability to measure adherence after adoption. Since we did not control the timing of DPP interactions after completion of the CT simulation scan procedures between the patient and care providers (ie, patients with issues would initiate symptom reporting to care providers on an as-needed basis), we could not accurately measure DPP adherence after adoption.

The principal barrier to DPP adoption identified in this study, poor ability to use smartphones, is a potentially modifiable barrier, and as such, plans are underway to develop a custom intervention geared at addressing this issue.

In conclusion, among patients with prostate cancer undergoing radical radiotherapy, poor self-reported ability to use smartphone technology was identified as the primary barrier to DPP adoption. Interventions geared at mitigating this barrier to DPP adoption are needed.

## Data Availability

The data sets generated during and/or analyzed during the current study are available from the corresponding author upon reasonable request.
